# Measuring up: the need for new assays for factor (F)XI/FXIa inhibitor therapies

**DOI:** 10.1016/j.rpth.2026.106640

**Published:** 2026-05-12

**Authors:** David Gailani, Melissa F. Glasner, Rasha Al-Lamee, María Cecilia Bahit, Jonathan M. Coutinho, S. Claiborne Johnston, Carolyn S.P. Lam, Tzu-Fei Wang, Wolfgang C. Winkelmayer, Jeffrey I. Weitz

**Affiliations:** 1 Vanderbilt University Medical Center, Nashville, Tennessee, USA; 2 The France Foundation, Old Lyme, Connecticut, USA; 3 Imperial College Healthcare NHS Trust and Imperial College London, London, UK; 4 Department INECO Neurociencias, Rosario, Argentina; 5 Amsterdam University Medical Center, Amsterdam, The Netherlands; 6 University of California, San Francisco, California, USA; 7 National Heart Centre Singapore, Duke-National University, Singapore; 8 University of Ottawa, The Ottawa Hospital, Ottawa, Ontario, Canada; 9 Baylor College of Medicine, Houston, Texas, USA; 10 Hamilton Health Sciences and McMaster University, Hamilton, Ontario, Canada

**Keywords:** activated partial thromboplastin time, anticoagulation, blood coagulation tests, direct oral anticoagulants, factor XI

## Abstract

**Background:**

As factor (F)XI/FXIa inhibitors advance through clinical development, a central challenge is determining how to assess their anticoagulant effects in routine practice.

**Laboratory assessment:**

Assays such as the activated partial thromboplastin time and FXI activity tests demonstrate dose-dependent changes with these agents but are limited by reagent variability, poor standardization, and weak correlation with bleeding risk. No Food and Drug Administration–approved or standardized assay currently exists to reliably quantify FXI/FXIa inhibition or guide clinical decision making.

**Clinical implications:**

Available clinical data suggest FXI/FXIa inhibitors provide robust antithrombotic efficacy with low bleeding risk, reducing the need for routine monitoring compared with traditional anticoagulants. However, patients often exhibit markedly prolonged activated partial thromboplastin time values that may be misinterpreted in acute settings, especially during surgery or episodes of bleeding. Conventional assays lack reliability for guiding care.

**Future directions:**

Developing standardized monitoring strategies and institutional protocols will be critical to support safe integration of these agents, ensure consistency across care settings, and guide management in special clinical situations.

## Essentials

• Factor (F)XI/FXIa inhibitors may reduce thrombosis with less impact on hemostasis than current agents.
• Assessing anticoagulant effects and interpreting assays remains a key clinical challenge.
• Standard assays show variable effects and do not reliably reflect bleeding risk or drug levels.
• Routine monitoring may be unnecessary, but targeted testing may guide care in select settings.

## 1 Introduction

Clinicians routinely prescribe anticoagulants for patients with or at risk of thromboembolism, including those with deep vein thrombosis, pulmonary embolism, atrial fibrillation (AF), recent surgery, or a history of blood clots [[1], [2], [3], [4], [5], [6]]. Current anticoagulants—which include vitamin K antagonists, unfractionated and low-molecular-weight heparins, oral factor (F)Xa inhibitors, and oral and parenteral thrombin inhibitors—are highly effective for managing most thrombotic disorders [2,3,[6], [7], [8]]. However, these agents achieve their therapeutic effects by targeting proteases, particularly FXa and thrombin, which are central to hemostasis at a site of injury [[9], [10], [11]]. The effects of most anticoagulants are dose dependent, and their impact on hemostasis increases the risk of serious bleeding [10,11].

Treatment with warfarin and heparin is monitored regularly with laboratory testing to maintain drug effects within a therapeutic range while reducing bleeding risk as much as possible [12,13]. Direct oral anticoagulants (DOACs) targeting thrombin or FXa cause less bleeding than warfarin and infrequently require monitoring with drug levels [14]. However, these agents can still cause life-threatening hemorrhage [15,16]. As a consequence, physicians may avoid prescribing them or prescribe suboptimal doses for patients who may otherwise benefit from therapy [[17], [18], [19]]. There is an unmet need for anticoagulants that effectively treat or prevent thrombosis while having a smaller impact on hemostasis than current drugs. Agents targeting the plasma protein FXI and its active protease, FXIa, are being investigated for this purpose.

## 2 Factor XI and Hemostasis

Factor XI is a dimer of identical 80-kDa subunits, each comprising 4 apple domains (A1-A4) and a trypsin-like catalytic domain (Figure 1) [[20], [21], [22]]. Factor XI is converted to FXIa by cleavage of the Arg^369^–Ile^370^ bond on at least 1 subunit (Figure 1) [21,22]. Factor XIIa and thrombin catalyze the conversion of FXI to FXIa, and other proteases may contribute to the process [[20], [21], [22], [23]]. In the original models of plasma coagulation based on the cascade/waterfall hypotheses [24,25], FXI is a component of the intrinsic pathway that drives contact activation-initiated clotting in the activated partial thromboplastin time (aPTT) assay used in clinical laboratories (Figure 2). Accordingly, complete FXI deficiency causes marked prolongation of the aPTT but not the prothrombin time assay, which is triggered through the extrinsic pathway (Figure 2). Indeed, the degree of aPTT prolongation caused by the absence of FXI is like or even greater than that caused by the complete deficiency of FVIII or FIX, the intrinsic pathway proteins missing in the severe bleeding disorders hemophilia A and hemophilia B, respectively. However, despite this, FXI deficiency causes milder and less consistent bleeding than is seen in patients with hemophilia [[26], [27], [28], [29]]. The significant soft tissue and joint bleeds characteristic of hemophilia are rarely observed in patients lacking FXI. These observations suggest that FXI’s contribution to the aPTT assay does not accurately reflect its physiological role in hemostasis. Factor XI–deficient patients may experience excessive trauma- or surgery-induced bleeding, particularly when the nasopharynx, oral cavity, or urinary tract is involved [[27], [28], [29]]. Factor XI–deficient women may also experience heavy menstrual bleeding [29]. However, in general, there is poor correlation between plasma FXI levels determined with aPTT assays and bleeding propensity [[29], [30], [31], [32]].

**Figure 1:**
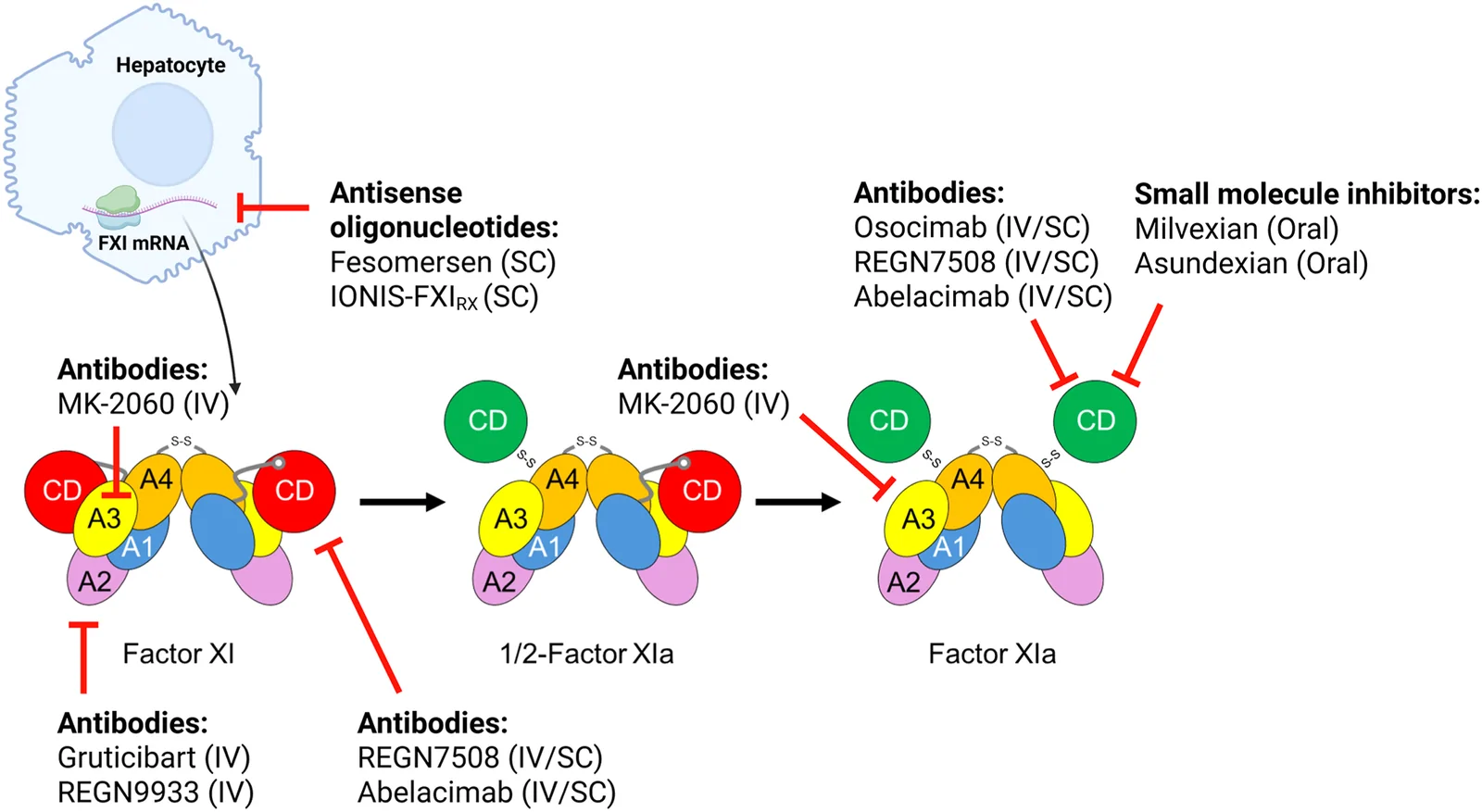
Mechanisms of action of drugs targeting factor (F)XI and FXIa. The FXI diagrams shown are based on the FXI crystal structure reported by Papagrigoriou et al [21]. The FXI molecule contains 2 identical subunits, each with 4 apple domains (A1-A4) and a catalytic domain (CD). The dimer is held together by interactions between the A4 domains. A FXI subunit is converted to an FXIa subunit by cleavage of the Arg^369^–Ile^370^ bond. Initially, 1 subunit is activated generating 1/2FXIa, followed by activation of the second subunit to form FXIa [22]. The FXI catalytic domain is in an inactive conformation (red) that is converted to an active conformation (green) after cleavage of the Arg^369^–Ile^370^ bond. The antisense oligonucleotides IONIS-FXI_RX_ and fesomersen are taken up by hepatocytes and form heteroduplexes with FXI mRNA, leading to RNAse-mediated mRNA degradation and reduced FXI synthesis. Several antibodies bind to both zymogen FXI and FXIa. Abelacimab and REGN7508 bind to the catalytic domain near the active site, locking it in an inactive state. MK-2060 binds to the A3 domain, preventing FXI activation by FXIIa and blocking FIX binding to FXIa. Gruticibart and REGN9933 bind to the A2 domain, inhibiting FXI activation during contact activation. Osocimab binds to the active site of FXIa, but not FXI, blocking its catalytic activity. The small molecules asundexian and milvexian bind within the FXIa active site, also blocking FXIa catalytic activity. IV, intravenous; SC, subcutaneous.

**Figure 2:**
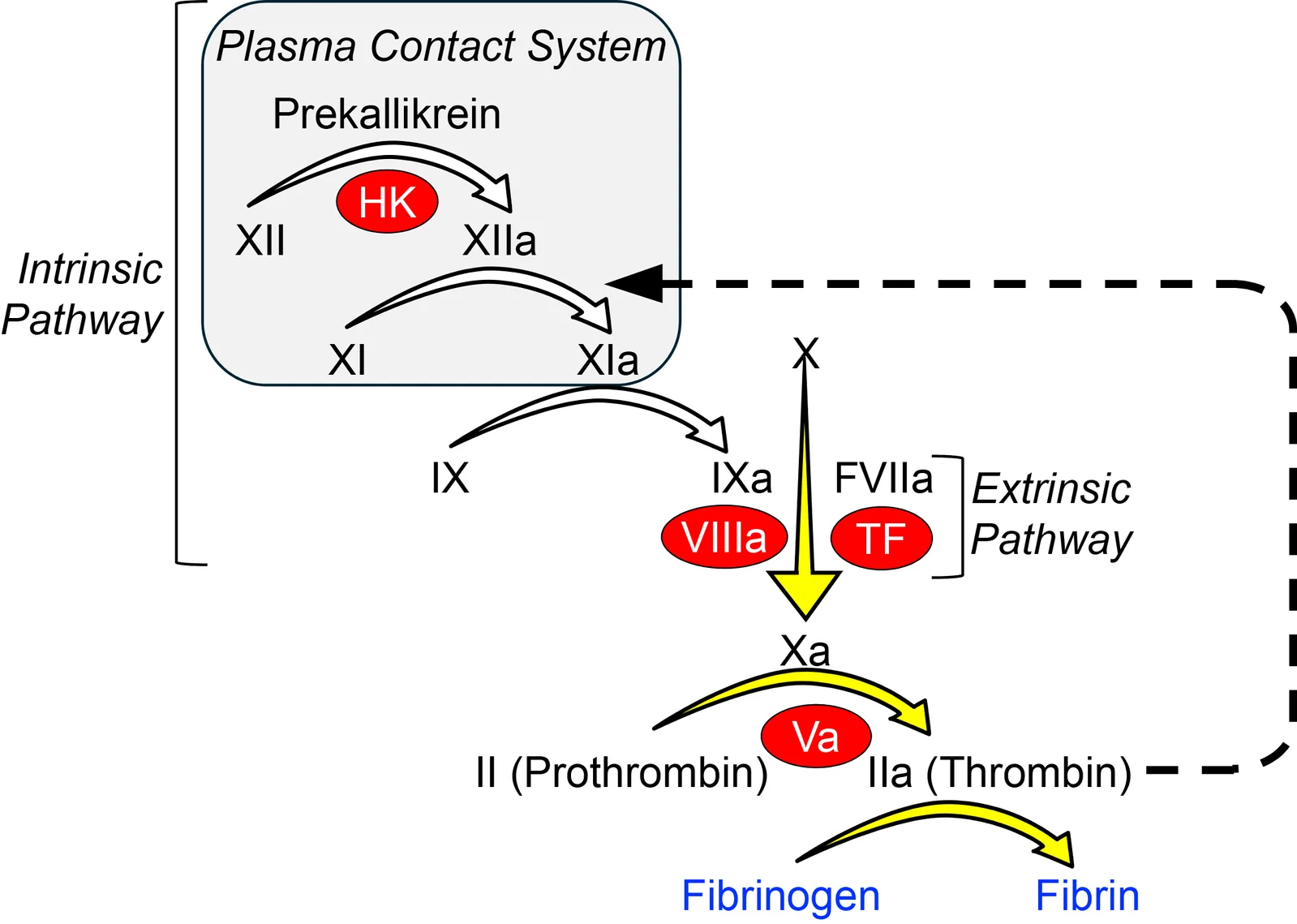
The coagulation cascade and the activated partial thromboplastin time (aPTT) assay. Shown is a model for fibrin formation used in most clinical coagulation laboratories. The process of thrombin generation involves sequential conversion of plasma zymogens (Roman numerals in black lettering) to their protease forms (indicated by a lower case “a”). A group of protein cofactors (red ovals, high-molecular-weight kininogen [HK], factor [F]VIII and FV, and tissue factor [TF]) facilitate these reactions. In the aPTT assay, thrombin generation is initiated by activating the plasma contact system (gray rectangle), setting off a series of reactions referred to as the intrinsic pathway of coagulation (white arrows). The prothrombin time assay is initiated by adding tissue factor to plasma, which initiates thrombin generation through the extrinsic pathway of coagulation. The intrinsic and extrinsic pathways both activate FX in the common pathway of coagulation (yellow arrows), leading to thrombin generation and conversion of soluble fibrinogen to insoluble fibrin clot (blue). Factor XI (XI) is a component of the intrinsic pathway that is converted to FXIa by FXIIa the aPTT assay. Factor XI is not involved in the prothrombin assay. In newer models of thrombin generation, FXI is the only component of the plasma contact system required for hemostasis. In these models, it is assumed that FXI is activated by a protease other than FXIIa, possibly thrombin (dashed line). In contrast to the aPTT assay, FXI is not part of the process that initiates thrombin generation in the newer models. Instead, it participates in sustaining thrombin generation after a fibrin clot has already formed. The contact factor, prekallikrein, which is part of the plasma contact system, is the precursor of the protease plasma kallikrein. Plasma kallikrein is structurally similar to FXIa, and the small-molecule FXIa inhibitors asundexian and milvexian inhibit both proteases. IV, intravenous; SC, subcutaneous.

In current models of thrombin generation, the primary activator of FIX is the FVIIa/tissue factor complex (Figure 2, the extrinsic pathway) [33,34]. In these models, FXIa is an ancillary contributor to FIX activation, rather than a driver of initial clot formation, as in the aPTT [23,35]. Factor XI activation occurs after initial thrombin generation and may be catalyzed by thrombin itself or by proteases other than FXIIa (Figure 2, dashed line). Models incorporating this functional reassignment of FXI are more compatible with the mild and variable phenotype of FXI deficiency and explain why some patients with severe FXI deficiency do not experience abnormal bleeding. In such individuals, the FVIIa/tissue factor complex may compensate for the lack of FXI.

## 3 Factor XI and Thrombosis

The proposed supportive role of FXI in hemostasis might suggest that its contributions to thrombosis would be relatively small. However, data from multiple epidemiological and genetic studies indicate that high plasma levels of FXI increase the risk of venous thromboembolism, ischemic stroke, and myocardial infarction [34,36,37], while low FXI levels reduce the risk of venous thromboembolism and ischemic stroke [[38], [39], [40]]. Furthermore, as part of the plasma contact system (Figure 2, gray rectangle), FXI may contribute to thrombus formation in settings where blood is in contact with nonbiological surfaces during procedures, such as cardiopulmonary bypass, extracorporeal membrane oxygenation, and renal dialysis, and in patients with mechanical heart valves, or central venous catheters [[41], [42], [43]].

The contribution of FXI to thrombosis and the relatively mild bleeding phenotype associated with severe FXI deficiency suggest that inhibiting FXI or FXIa may produce an antithrombotic effect with less disruption of hemostasis than current anticoagulants [44,45]. In particular, from a safety standpoint, clinical observations in patients with severe FXI deficiency suggest that drugs targeting this protein would not increase the risk of intracranial bleeding, which is responsible for a substantial number of treatment-related deaths with warfarin and the DOACs. Several important issues regarding the use of anticoagulants targeting FXI and FXIa remain unresolved. In this section, we focus on the need for specific laboratory methods to detect these drugs and measure their effects.

## 4 Inhibiting FXI and FXIa for Therapeutic Purposes

Several agents that reduce FXI synthesis, FXI activation, and/or FXIa activity have been tested or are being tested in phase 2 and phase 3 trials (Table) [[46], [47], [48], [49], [50], [51], [52], [53], [54], [55], [56], [57], [58], [59], [60], [61], [62], [63], [64], [65], [66], [67], [68], [69], [70]] . These include orally administered small molecules that reversibly block the FXIa active site (asundexian and milvexian) [60,67], antibodies that bind to specific domains on FXI or FXIa (abelacimab, osocimab, gruticibart [also known as AB023 or xisomab], REGN7508, REGN9933, and MK-2060) [48,50,53,54,56], and antisense oligonucleotides (ASOs) that induce FXI mRNA degradation in the liver (IONIS-FXI_Rx_ and fesomersen) (Figure 1) [46,47]. Phase 2 trials were conducted in individuals undergoing total knee arthroplasty [46,49,53,57,68] and in patients with AF [58,61], acute ischemic stroke [62,69], acute myocardial infarction [63], catheter-induced thrombosis [51], or end-stage kidney disease [52,55,64]. Factor XI/FXIa reduction or inhibition after knee replacement reduced venous thrombus formation, as well as or better than the standard of care, with a low incidence of bleeding [46,49,54,57,68]. In patients with ischemic stroke, phase 2 and preliminary phase 3 data suggest oral FXIa inhibitors reduce the incidence of recurrent symptomatic stroke [62,63,69]. In the Phase 3 OCEANIC-STROKE trial (Oral faCtor Eleven A iNhibitor asundexian as novel antithrombotiC for secondary stroke prevention), asundexian reduced the risk of recurrent ischemic stroke by 26% relative to placebo, without increasing the rate of bleeding in patients at risk of secondary stroke who were receiving antiplatelet therapy [65]. Phase 3 studies in patients with atrial fibrillation (LIBREXIA-AF [NCT05757869] and LILAC [A study to evaLuate the effIcacy and Safety of abeLacimab in High-risk Patients With Atrial Fibrillation Who Have Been Deemed Unsuitable for Oral antiCoagulation]-TIMI 76, [NCT05712200], or for secondary stroke prevention (LIBREXIA-STROKE [NCT05702034]) are underway. Based on phase 2 dose-finding data, the phase 3 studies were largely designed under the assumption that bleeding will be limited in patients on FXI/FXIa inhibitors, even at high levels (>90%) of inhibition. While antibodies targeting FXI or FXIa have long-lasting effects (weeks to months) and are not readily reversible in the event of bleeding, most FXI-deficient patients with bleeding can be treated with approaches that do not require FXI replacement, such as recombinant-activated FVII and tranexamic acid [[71], [72], [73]]. The use of recombinant FVIIa to counteract FXI inhibition should be used with caution in patients who have preexisting thrombotic risk factors, due to rare but serious adverse events [74]. Nevertheless, it is anticipated that such approaches will also be useful for treating patients with bleeding on FXI/FXIa inhibitors [[73], [74], [75]].

**Table:** Overview of FXI/XIa inhibitors.

Drug	Phase 2 agents						Phase 3 AGENTS			
	IONIS-FXI_Rx_	Fesomersen	Osocimab	Gruticibart	REGN7508	MK-2060	Abelacimab	Asundexian	Milvexian	REGN9933
References	46	47	48,49	50–52	53	54,55	56–59	60–66	67–70	53
Agent class	Antisense oligonucleotide	Antisense oligonucleotide	Fully human mAB IgG1	Humanized mAB IgG1	Humanized mAB	Fully human mAB IgG1	Fully human mAB IgG1	Small-molecule active site inhibitor	Small-molecule active site inhibitor	Humanized mAB
Mechanism of action	Binds to FXI mRNA in hepatocytes causing its degradation and reduced FXI synthesis	Binds to FXI mRNA in hepatocytes causing its degradation and reduced FXI synthesis	Binds to FXIa catalytic domain, blocking interactions with substrates	Binds to FXI A2 domain, interfering with FXI activation by FXIIa	Binds to FXI and FXIa catalytic domain blocking substrates interaction	Binds to FXI A3 domain, blocking FXI activation by FXIIa and FIX activation by FXIa	Binds to FXI and FXIa catalytic domain, locking them in a zymogen conformation	Blocks access to the FXIa protease active site	Blocks access to the FXIa protease active site	Binds to FXI A2 domain, interfering with FXI activation by FXIIa
Route of administration	SC	SC	IV or SC	IV	IV or SC	IV	IV or SC	Oral	Oral	IV
Time to peak drug concentration	∼6 h	1.5-2.5 h	1-4 h IV, 4-6 d SC	6-42 min	Data unavailable	Data unavailable	1 h IV, 7-21 d SC	1-4 h	∼3 ho	Data unavailable
Effect on aPTT	Dose-dependent prolongation	Dose-dependent prolongation	Dose-dependent prolongation	Reaches maximum when IgG saturates FXI	Data unavailable	Reaches maximum when IgG saturates FXI	Reaches maximum when IgG saturates FXI	Dose-dependent prolongation	Dose-dependent prolongation	Reaches maximum when IgG saturates FXI
Time to peak aPTT	3-12 wk	15-30 d	∼2 h IV	10-30 min	Data unavailable	Data unavailable	1 h to 5 d depending on route	4-8 h	2-4 h	∼8 h
aPTT reagent in phase 1/2 studies	Actin FSL	Actin FSL	Kaolin	Silica	Silica	Data unavailable	Actin FSL	Silica or kaolin	Silica	Silica
FXI activity by 1-stage aPTT assay	Value should represent the true residual plasma FXI activity level	Value should represent the true residual plasma FXI activity level	Cannot be used to accurately assess drug activity	Interferes with the activation of FXI by FXIIa, and the result may not reflect the true residual FXI activity	Inversely correlated with drug concentration up to saturation	Probably inversely correlated with drug concentration up to saturation	Inversely correlated with drug concentration up to saturation	Results are unlikely to accurately reflect the degree of FXIa inhibition by the drug	Results are unlikely to accurately reflect the degree of FXIa inhibition by the drug	Interferes with the activation of FXI by FXIIa, and the result may not reflect true residual FXI activity
Off-target effect on aPTT	Possible that the ASO may reduce the synthesis of a protein other than FXI that is required for the aPTT, but not observed	Possible that the ASO may reduce the synthesis of a protein other than FXI that is required for the aPTT, but not observed	Should be specific for FXIa	Should be specific for FXI and FXIa	Should be specific for FXI and FXIa	Should be specific for FXI and FXIa	Should be specific for FXI and FXIa	Inhibits the protease plasma kallikrein at high drug concentrations	Inhibits the protease plasma kallikrein at high drug concentrations	Should be specific for FXI and FXIa

aPTT, activated partial thromboplastin time; ASO, antisense oligonucleotide; F, factor; IV, intravenous; SC, subcutaneous.

## 5 The Need to Monitor FXI/FXIA Inhibitors with Laboratory Tests

A method for measuring an anticoagulant or its effect could be used (1) to establish that a therapeutic drug level has been achieved and/or (2) to keep the drug level below a concentration in which bleeding becomes excessive. At this point, no need for testing has been established for these or other indications for FXI/FXIa inhibitors.

The AZAELEA (A Multicenter, RandomiZed, Active-ControLled Study to Evaluate the Safety and Tolerability of Two Blinded Doses of Abelacimab Compared With Open-Label Rivaroxaban in Patients With Atrial Fibrillation) TIMI-71 trial compared the anti-FXI/FXIa antibody abelacimab and the FXa-inhibiting DOAC rivaroxaban in patients with AF with a moderate to high bleeding risk [58]. Patients receiving abelacimab had a >98% reduction in FXI activity over a mean follow-up period of 2.1 years. The incidence of major or clinically relevant nonmajor bleeding was significantly lower for abelacimab (0.99 and 1.22 events/100 person-years, for 90 mg and 150 mg doses, respectively) than for rivaroxaban (3.73 events/100 person-years). About one-third of patients underwent at least 1 invasive procedure during the study [59]. For patients undergoing a procedure within 30 days of receiving a dose of abelacimab, the incidence of major or clinically relevant nonmajor bleeding was only 0.9%. In most cases, no treatment to reverse the effect of the abelacimab was given prior to surgery. Taken as a whole, these observations suggest that dosing regimens that achieve near maximum inhibition of FXI/FXIa are generally well tolerated and that testing to establish that drug concentrations are within a therapeutic window is not generally needed. However, three-quarters of the procedures in the AZAELEA TIMI-71 study were considered low risk for bleeding, and few involved high-risk tissues such as the oropharynx and urinary tract [59]. Furthermore, prophylactic use of tranexamic acid in some FXI-inhibitor–treated patients may have contributed to the low observed bleeding rates, highlighting the need for further studies to clarify bleeding risk in different surgical settings. There may be scenarios where specific types of surgery, comorbidities, and/or combinations of anticoagulants will require adjustments of an FXI/XIa inhibitor. Accurate methods for assessing drug effects and/or measuring drug concentrations would facilitate these adjustments.

## 6 Methods for Measuring the Effects of FXI/FXIA Inhibitors

The most widely available test for assessing FXI activity is the standard aPTT assay. Most phase 1 and 2 trials of FXI/FXIa inhibitors included aPTT data (Table). The aPTT is not standardized, as a variety of reagents and automated platforms are used for testing. The assay is also relatively insensitive to mild to moderate FXI deficiency (FXI activity of 30%-50% of normal) [[76], [77], [78]]. However, the high degree of targeted inhibition in most clinical trials significantly prolonged the aPTT (ratios of patient aPTT: normal aPTT > 1.5) [46,[48], [49], [50],^53^,^54^,^57^,^68^]. For a given aPTT assay within a study, the extent of prolongation in response to therapy is generally dose or concentration dependent (Table) [46,[48], [49], [50],^53^,^54^,^57^,^68^]. However, as when using the aPTT to monitor parenteral anticoagulants such as unfractionated heparin, bivalirudin, and argatroban, assay limitations must be considered. We can expect that the lack of standardization will result in a range of assay sensitivities to FXI/FXIa inhibitors between institutions [77,78]. Individual patient-specific factors such as underlying coagulopathies, inflammation, and poor liver function will affect the aPTT independently of drug activity [79,80] and are likely to produce synergistic or additive effects with a drug targeting FXI/FXIa, which will confound interpretation.

aPTT assays will respond differently to drugs targeting FXI or FXIa depending on their mechanism of action. For some drugs, aPTT results should reflect reductions in plasma FXI activity (or free FXI activity) in a manner similar to the detection of reduced activity in inherited FXI deficiency. Drugs that reduce FXI synthesis (ASOs and siRNAs) and antibodies that produce the equivalent of a FXI-deficient state by locking the zymogen in an inactive or unactivatable conformation (abelacimab, REGN7508, and MK-2060) fall into this category [46,53,56]. Antibodies to the FXI A2 domain (gruticibart and REGN9933) reduce FXI activation by FXIIa but do not inhibit FXIa activation of FIX [53,81]. These drugs may have smaller effects on the aPTT than REGN7508 and MK-2060, and extrapolating from the absolute aPTT value to a specific level of inhibition will be difficult. Assuming high specificity (no off-target drug effects), FXI antibodies should raise the aPTT to a plateau value corresponding to target saturation [82]. However, the aPTT values at the plateau may vary between antibodies and with different aPTT assays. For ASOs and siRNAs, FXI activity as a percentage of a normal plasma control can be measured using a 1-stage aPTT-based FXI assay. Since ASOs and siRNAs reduce FXI protein in plasma, measuring the residual FXI antigen with an antibody-based ELISA is another option. The 1-stage FXI assay may also provide an estimate of the degree of residual free FXI activity (ie, FXI not bound to antibody) in patients on abelacimab, REGN7508, and MK-2060.

The situation is more complex for drugs that interact specifically with the FXIa active site (asundexian, milvexian, and osocimab) [48,60,67]. Here, FXI must first be converted to FXIa. The inhibitors then neutralize FXIa with various on- and off-rates while competing with plasma FXIa substrates. The aPTT is a rate-based assay, and drugs that inhibit FXIa more rapidly will tend to produce the greatest aPTT prolongation. However, observations of the effects of DOACs on PT and aPTT assays suggest that differences in clotting times do not necessarily reflect differences in clinical efficacy. This limitation will likely hold for FXIa inhibitors. Nonclinical and clinical studies with asundexian and milvexian consistently showed concentration-dependent aPTT prolongation [[61], [62], [63], [64],[66], [67], [68],^70^,[83], [84], [85], [86]]. However, the extent of prolongation varied depending on the aPTT reagent used, with 1 study reporting high variability in the plasma concentration of asundexian required to prolong different aPTT assays [86]. Another study showed, as expected, that abelacimab caused aPTT prolongation that plateaued at a value consistent with >98% reduction of FXI activity [82]. However, there was no plateau with asundexian or milvexian, and at higher drug concentrations, the aPTT increased to values in excess of those expected with complete FXI deficiency. This likely reflects off-target inhibition of plasma kallikrein, the activated form of prekallikrein (Figure 2), which is a homolog of FXIa [82]. Cumulatively, these issues indicate that the aPTT will not be useful for adjusting FXIa active site inhibitors, much as it is of limited value in patients on DOACs directed at FXa or thrombin. Similarly, FXI activity measured by 1-stage aPTT-based factor assays or antibody-based assays of free FXI antigen is best suited for situations where there is a true reduction in circulating free FXI protein and would not be useful for monitoring FXIa active site inhibitors.

For inhibitors that only interact with FXIa (asundexian, milvexian, and osocimab), an assay that adds a fixed amount of FXIa to drug-containing plasma, then measures residual FXIa activity with a chromogenic or fluorogenic substrate, may be the best approach [87]. Assays based on inhibition of exogenously added FXa or thrombin are widely used to measure plasma concentrations of heparin-like inhibitors (unfractionated and low-molecular-weight heparin and fondaparinux) and have been adapted for measuring thrombin and FXa active site inhibitors such as bivalirudin and the DOACs [[88], [89], [90], [91]]. An assay based on FXIa inhibition was used to measure the effect of asundexian in plasma in phase 2 clinical trials [60,66,85]. However, this assay used FXIa generated from endogenous plasma FXI by inducing contact activation, and the amount of FXIa in the system is, therefore, unknown and likely to vary between samples [61,66]. A system that uses exogenous FXIa may improve the assay’s accuracy and reproducibility.

## 7 Discussion

Trials of FXI/XIa inhibitors consistently show favorable safety profiles, with a low risk of severe bleeding even at doses that produce near complete target inhibition [46,49,53,[57], [58], [59],^61^,^62^,^69^]. This is unlike anticoagulants targeting FXa or thrombin, where complete inhibition of a target would cause unacceptable bleeding. The risk of spontaneous intracranial bleeding or severe gastrointestinal hemorrhage in patients with inherited total FXI deficiency appears to be similar to that of the general population, and results of clinical trials of FXI/FXIa inhibitors are consistent with these observations. Thus, therapy targeting FXI/FXIa could be designed to negate all or nearly all FXI activity, and routine laboratory monitoring to maintain drug concentrations within a therapeutic range may not be necessary for most patients. As FXI- and FXIa-targeted therapies advance through clinical trials, the central issue may not be the establishment of a general monitoring system, but rather the identification of scenarios in which testing will aid patient management.

We need to identify clinical situations in which FXI/FXIa inhibitors promote bleeding. These may include major surgery and procedures involving tissues with high-fibrinolytic activity, procedures in patients with prior abnormal bleeding or a known coagulopathy, concurrent use of other anticoagulants or antiplatelet agents, or situations where comorbidities such as liver disease are present. If superimposed on other anticoagulants, such as heparin, which also increases the aPTT, this effect may confound laboratory monitoring and create uncertainty regarding the magnitude of anticoagulation. In these situations, targeted laboratory assessment may help guide procedure timing, perioperative planning, and supportive care strategies.

From a laboratory standpoint, as >1 type of FXI/FXIa inhibitor may become available, we will need to become familiar with their mechanisms of action. Agents that reduce circulating free FXI protein (ASOs or FXI-sequestering antibodies) may be best assessed with 1-stage FXI activity or antigen-based assays, whereas active-site FXIa inhibitors may require an assay that directly measures enzymatic inhibition. Initially, such assays may only be available at larger centers. In smaller hospitals and clinics where the aPTT is the only test available, the sensitivity of the particular aPTT assay to a specific FXI/FXIa inhibitor may need to be established. Clinicians should work with their colleagues in hospital laboratories to understand which assays are available locally, how they are performed, and how results should be interpreted. Establishing institutional pathways for selective FXI/XIa testing, rather than ad hoc ordering, may reduce misinterpretation and unnecessary interventions.

Finally, clinical judgment will remain paramount. Until standardized assays and outcome-linked thresholds are established, laboratory results should be interpreted in conjunction with bleeding history, procedural risk, and overall clinical context rather than used as absolute decision points.
