# Supplementary material for: Measuring up: the need for new assays for factor (F)XI/FXIa inhibitor therapies
Source: Res Pract Thromb Haemost. 2026 May 12;10(Suppl 3):106640. doi: 10.1016/j.rpth.2026.106640 (PMC13700368; doi:10.1016/j.rpth.2026.106640)
Supplement: Supplementary file 1 [file mmc1.docx]

ONLINE ACTIVITY INTRODUCTION (CME/CE) TEXT

| Activity Title | Measuring Up: The Need for New Assays for FXI/FXIa Inhibitor Therapies |
| --- | --- |
| Topic | **Factor XI and XIa** |
| Accreditation Type | **Physicians**—maximum of 1.0 *AMA PRA Category 1 Credit(s)*™ |
| Accreditor | The France Foundation |
| Release Date: | November 1, 2025 |
| Expiration Date: July 14, 2026 | October 31, 2026 |
| Estimated Time to Complete Activity | **1.0 hour** |

**TARGET AUDIENCE**

The primary target audiences for this initial education include health care providers (physicians, nurse practitioners, and PAs) specializing in hematology, cardiology, neurology, and nephrology.

**PROGRAM OVERVIEW**

The aim of this educational program is to enhance clinicians' knowledge and confidence about new assays for FXI/FXIa inhibitor therapies.

**Please review this information, and proceed below**

**Learning Objectives**

- Examine how differing pharmacodynamic assays impact efficacy interpretation across FXI/FXIa studies
- Assess the utility and limitations of coagulation assays in monitoring and optimizing FXI/FXIa inhibitor therapy in practice

**Faculty**

**CORRESPONDING AUTHOR:**

**Melissa F. Glasner, MSc**

Manager, Medical Content

The France Foundation

Old Lyme, Connecticut

**LEAD AUTHOR:**

**David Gailani, MD**

Professor of Pathology, Microbiology, and Immunology

Ernest W. Goodpasture Chair in Experimental Pathology for Translational Research

Professor of Medicine

Nashville, Tennessee

**Jeffrey Weitz, MD (Chair)**

Professor of Medicine, Biochemistry, Biomedical Sciences

Hamilton Health Sciences and McMaster University

Hamilton, Ontario, Canada

**Rasha Al-Lamee, MD, PhD, BSc**

Interventional Cardiology

Consultant at Imperial College Healthcare NHS

Trust and Clinical Academic

Imperial College

London, England

**María Cecilia Bahit, MD**

Chief of Cardiology, Department INECO Neurociencias

Co-Chair World Stroke Organization Heart and Brain Task Force

Rosario, Argentina

**Jonathan Coutinho, MD, PhD, FESO**

Professor, Neurology

Amsterdam University Medical Centers

Department of Neurology

Amsterdam, The Netherlands

**David Gailani, MD**

Professor of Pathology, Microbiology, and Immunology

Ernest W. Goodpasture Chair in Experimental Pathology for Translational Research

Professor of Medicine

Nashville, Tennessee

**Clay Johnston, MD, PhD**

CMO, Harbor Health

Dell Medical School

UT-Austin

University of Texas

Austin, Texas

**Carolyn S.P. Lam, MD, PhD**

Senior Consultant

Department of Cardiology and Director of Women's Heart Health

National Heart Centre Singapore

Duke-National University

Singapore

**Tzu-Fei Wang, MD, MPH**

Associate Professor, Division of Hematology

University of Ottawa

The Ottawa Hospital, General Campus

Ottawa, Ontario, Canada

**Wolfgang C. Winkelmayer, MD, MPH, ScD, FASN**

Gordon A. Cain Professor of Medicine

Chief, Section of Nephrology

Director, Selzman Institute for Kidney Health

Baylor College of Medicine

Houston, Texas

## INSTRUCTIONS FOR PARTICIPATION AND REQUESTING CREDIT

There are no fees for participating and receiving credit for this activity. Successful completion is achieved by reviewing the CE information, completing the activity, and completing the posttest and evaluation form.

**Credit Fulfillment**

- If you are requesting AMA credits or a certificate of participation, your certificate will be available for download

**ACCREDITED PROVIDER**

This activity is jointly provided by The France Foundation and the International Society on Thrombosis and Haemostasis.

**Accreditation Statement**

In support of improving patient care, this activity has been planned and implemented by The France Foundation (TFF) and the International Society on Thrombosis and Haemostasis (ISTH). The France Foundation is jointly accredited by the Accreditation Council for Continuing Medical Education (ACCME), the Accreditation Council for Pharmacy Education (ACPE), and the American Nurses Credentialing Center (ANCC) to provide continuing education for the health care team.

## Physician Credit Designation

The France Foundation designates this enduring activity for a maximum of 1.0 AMA PRA Category 1 Credit(s)™. Physicians should claim only the credit commensurate with the extent of their participation in the activity.

All other health care professionals completing this course will be issued a statement of participation.

**DISCLOSURE POLICY**

In accordance with the ACCME Standards for Integrity and Independence in Accredited Continuing Education, The France Foundation (TFF) and International Society on Thrombosis and Haemostasis (ISTH) require that individuals in a position to control the content of an educational activity disclose all relevant financial relationships with any commercial entity. TFF and ISTH resolve all conflicts of interest to ensure independence, objectivity, balance, and scientific rigor in all their educational programs.

Furthermore, TFF and ISTH seek to verify that all scientific research referred to, reported, or used in a CME/CE activity conforms to the generally accepted standards of experimental design, data collection, and analysis. TFF and ISTH are committed to providing learners with high-quality CME/CE activities that promote improvements in health care and not those of a commercial interest.

Planning committee members, faculty, reviewers, and activity staff have disclosed the following relevant financial relationships. All relevant financial relationships listed have been mitigated.

| Name of Individual | Role in Activity | Name of Commercial Interest(s) | Nature of Relationship(s) | Mechanism(s) implemented to resolve conflict of interest |
| --- | --- | --- | --- | --- |
| Rasha Al-Lamee, MD, PhD, BSc | Planning Committee member | 1. Philips, Abbott Vascular, Medtronic, Menarini, Omniprex 2. CathWorks, Janssen Pharmaceuticals | 1. Speaker honorarium 2. Advisory Board | All final planning decisions concerning content, learning objectives, and evaluation questions were made by the non-conflicted Planning Committee Member  Content was reviewed by a non-conflicted content reviewer to ensure that it is not commercially biased, is fair-balanced, and is based on scientific evidence and/or clinical reasoning |
| María Cecilia Bahit, MD | Planning Committee member | 1. Janssen/Johnson & Johnson, Anthos Therapeutics, BMS/Pfizer, Merck | 1. Academic consulting, committee work, membership on grant application review groups, and honoraria | All final planning decisions concerning content, learning objectives, and evaluation questions were made by the non-conflicted Planning Committee Member  Content was reviewed by a non-conflicted content reviewer to ensure that it is not commercially biased, is fair-balanced, and is based on scientific evidence and/or clinical reasoning |
| Jonathan Coutinho, MD, PhD, FESO | Planning Committee member | 1. Bayer, Siemens, AstraZeneca | 1. Contracted research | All final planning decisions concerning content, learning objectives, and evaluation questions were made by the non-conflicted Planning Committee Member  Content was reviewed by a non-conflicted content reviewer to ensure that it is not commercially biased, is fair-balanced, and is based on scientific evidence and/or clinical reasoning |
| David Gailani, MD | Planning Committee member, Faculty | 1. Anthos Therapeutics, Bayer Pharma, Bristol-Myers Squibb, Ionis, Janssen 2. Anthos Therapeutics, Bayer Pharma, Bristol-Myers Squibb, Ionis, Janssen | 1. Non-CE Consulting 2. Received honoraria and travel support from some of the listed companies | All final planning decisions concerning content, learning objectives, and evaluation questions were made by the non-conflicted Planning Committee Member  Content was reviewed by a non-conflicted content reviewer to ensure that it is not commercially biased, is fair-balanced, and is based on scientific evidence and/or clinical reasoning |
| Clay Johnston, MD, PhD | Planning Committee member | 1. Solay, Kisbee 2. Johnson & Johnson, BMS | 1. Non-CE consulting 2. Contract research | All final planning decisions concerning content, learning objectives, and evaluation questions were made by the non-conflicted Planning Committee Member  Content was reviewed by a non-conflicted content reviewer to ensure that it is not commercially biased, is fair-balanced, and is based on scientific evidence and/or clinical reasoning |
| Carolyn S.P. Lam, MD, PhD | Planning Committee member | 1. Alleviant Medical, Allysta Pharmaceutical, AnaCardio AB, Applied Therapeutics, AstraZeneca, Bayer, Biopeutics, Boehringer Ingelheim, Boston Scientific, Bristol Myers Squibb, CardioRenal, CPC Clinical Research, Eli Lilly, Hanmi, Impulse Dynamics, Intellia Therapeutics, Ionis Pharmaceutical, Janssen Research & Development LLC, Merck, Novartis, Novo Nordisk, ProSciento Inc, Quidel Corporation, Radcliffe Group Ltd., Recardio Inc, ReCor Medical, Roche Diagnostics, Sanofi, Siemens Healthcare Diagnostics, Us2.ai 2. Novo Nordisk, Roche Diagnostics 3. Us2.ai 4. Patent pending | 1. Academic consulting, membership on grant application review groups and honoraria 2. Contract Research 3. Stock ownership 4. Patent pending: PCT/SG2016/050217   Method for diagnosis and prognosis of chronic heart failure  US Patent No. 10,702,247  Automatic clinical workflow that recognizes and analyzes 2-dimensional and Doppler echo images for cardiac measurements and the diagnosis, prediction, and prognosis of heart disease | All final planning decisions concerning content, learning objectives, and evaluation questions were made by the non-conflicted Planning Committee Member  Content was reviewed by a non-conflicted content reviewer to ensure that it is not commercially biased, is fair-balanced, and is based on scientific evidence and/or clinical reasoning |
| Tzu-Fei Wang, MD, MPH | Planning Committee member | No relevant financial relationships | NA | NA |
| Wolfgang C. Winkelmayer, MD, MPH, ScD, FASN | Planning Committee member | 1. Akebia, Anthos, Ardelyx, AstraZeneca, Bayer,   Boehringer Ingelheim, Cadrenal,  GlaxoSmithKline, Natera, Novartis, Pharmacosmos, Unicycive, Vera   2. Bayer, Merck Sharpe Dohme | 1. Non-CE consulting      2. Contract research | All final planning decisions concerning content, learning objectives, and evaluation questions were made by the non-conflicted Planning Committee Member  Content was reviewed by a non-conflicted content reviewer to ensure that it is not commercially biased, was fair-balanced, and is based on scientific evidence and/or clinical reasoning |
| Jeffrey Weitz, MD | Planning Committee member | 1. Alnylam, Anthos, Bayer, Boehringer Ingelheim, Ionis, Daiichi-Sankyo, Bristol Myers Squibb, Johnson & Johnson, Merck, Novartis, Pfizer, Regeneron | 1. Non-CE Consulting | All final planning decisions concerning content, learning objectives, and evaluation questions were made by the non-conflicted Planning Committee Member  Content was reviewed by a non-conflicted content reviewer to ensure that it is not commercially biased, is fair-balanced, and is based on scientific evidence and/or clinical reasoning |
| Amanda Noe | TFF Staff | No relevant financial relationships | NA | NA |
| Cary Clark | ISTH Staff | No relevant financial relationships | NA | NA |
| Nathan Koenning | ISTH Staff | No relevant financial relationships | NA | NA |
| Debra Fernandez | TFF Staff | No relevant financial relationships | NA | NA |
| Heather Tarbox, MPH | TFF Staff | No relevant financial relationships | NA | NA |
| Melissa Glasner, MS | Planning Committee member, Faculty | No relevant financial relationships | NA | NA |
| Patrick Harty, PhD | Planning Committee member, TFF Content Reviewer | No relevant financial relationships | NA | NA |
| Miriam Giles | TFF Staff | No relevant financial relationships | NA | NA |

**Disclosure of Unlabeled Use:**

TFF and ISTH require CME faculty (speakers) to disclose when products or procedures being discussed are off label, unlabeled, experimental and/or investigational, and any limitations on the information that is presented, such as data that are preliminary or that represent ongoing research, interim analyses, and/or unsupported opinion. Faculty in this activity may discuss information about pharmaceutical agents that are outside of US Food and Drug Administration approved labeling.

This information is intended solely for continuing medical education and is not intended to promote off-label use of these medications. TFF and ISTH do not recommend the use of any agent outside of the labeled indications. If you have questions, contact the Medical Affairs Department of the manufacturer for the most recent prescribing information.

**COMMERCIAL SUPPORT ACKNOWLEDGMENT**

This activity is supported by an independent educational grant from Anthos Therapeutics and Bayer.

**Disclaimer**

The France Foundation and ISTH present this information for educational purposes only. The content is provided solely by faculty who have been selected because of recognized expertise in their field. Participants have the professional responsibility to ensure that products are prescribed and used appropriately on the basis of their own clinical judgment and accepted standards of care. The France Foundation, ISTH, and the commercial supporter(s) assume no liability for the information herein.

**CONTACT INFORMATION**

**If you have questions about this CME activity, please contact The France Foundation at 860-434-1650 or** [info@francefoundation.com](mailto:info@francefoundation.com)**.**

**COPYRIGHT INFORMATION**
Copyright © 2025 The France Foundation. Any unauthorized use of any materials on the site may violate copyright, trademark, and other laws. You may view, copy, and download information or software ("materials") found on the site subject to the following terms, conditions, and exceptions:

- The materials are to be used solely for personal, noncommercial, informational, and educational purposes. The materials are not to be modified. They are to be distributed in the format provided with the source clearly identified. The copyright information or other proprietary notices may not be removed, changed, or altered.
- Materials may not be published, uploaded, posted, or transmitted (other than as set forth herein) without The France Foundation's prior written permission

**PRIVACY POLICY**
The France Foundation protects the privacy of personal and other information regarding participants and educational collaborators. The France Foundation will not release personally identifiable information to a third party without the individual's consent, except when such information is required for reporting purposes to the ACCME.

The France Foundation maintains physical, electronic, and procedural safeguards that comply with federal regulations to protect against the loss, misuse, or alteration of information that we have collected from you.

Additional information regarding The France Foundation’s Privacy Policy can be viewed at <http://www.francefoundation.com/privacy>.

**CONTACT INFORMATION**

**If you have questions about this CME activity, please contact The France Foundation at 860-434-1650 or** [info@francefoundation.com](mailto:info@francefoundation.com)**.**

[CLICK](http://comp.realcme.com/cms/user?TB_iframe=true&width=655) **HERE TO BEGIN ACTIVITY**

**Please review this information, and proceed below**
